# What Is Medical Extended Reality? A Taxonomy Defining the Current Breadth and Depth of an Evolving Field

**DOI:** 10.1089/jmxr.2023.0012

**Published:** 2024-01-25

**Authors:** Brennan M.R. Spiegel, Albert Rizzo, Susan Persky, Omer Liran, Brenda Wiederhold, Susan Woods, Kate Donovan, Korak Sarkar, Henry Xiang, Sun Joo (Grace) Ahn, Rohan Jotwani, Min Lang, Margot Paul, Mike Senter-Zapata, Keith Widmeier, Haipeng Zhang

**Affiliations:** 1 Cedars-Sinai, Department of Medicine, Division of Health Services Research Virtual Medicine Program, Los Angeles, California, USA.; 2 Division of Gastroenterology, Cedars-Sinai Department of Medicine, Los Angeles, California, USA.; 3 Medical Virtual Reality Lab, University of Southern California Institute for Creative Technologies, Los Angeles, California, USA.; 4 Social and Behavioral Research Branch, National Human Genome Research Institute, Bethesda, Maryland, USA.; 5 Cedars-Sinai Department of Psychiatry and Behavioral Sciences, Los Angeles, California, USA.; 6 Virtual Reality Medical Center, San Diego, California, USA.; 7 Interactive Media Institute, San Diego, California, USA.; 8 Tufts School of Medicine, Boston, Massachusetts, USA.; 9 Boston Children's Hospital, Boston, Massachusetts, USA.; 10 Ochsner Health, New Orleans, Louisiana, USA.; 11 Veterans Affairs Administration, New Orleans, Louisiana, USA.; 12 Nationwide Children's Hospital, Colombus, Ohio, USA.; 13 Center for Advanced Computer-Human Ecosystems, University of Georgia, Athens, Georgia, USA.; 14 Weill Cornell Medicine, New York, New York, USA.; 15 Department of Radiology, Massachusetts General Hospital, Boston, Massachusetts, USA.; 16 Stanford University, Palo Alta, California, USA.; 17 Massachusetts General Brigham, Boston, Massachusetts, USA.; 18 Children's Hospital of Philadelphia, Philadelphia, Pennsylvania, USA.; 19 Harvard Medical School, Boston, Massachusetts, USA.

**Keywords:** medical extended reality (MXR), virtual reality, augmented reality, mixed reality, digital health, immersive technologies

## Abstract

Medical extended reality (MXR) has emerged as a dynamic field at the intersection of health care and immersive technology, encompassing virtual, augmented, and mixed reality applications across a wide range of medical disciplines. Despite its rapid growth and recognition by regulatory bodies, the field lacks a standardized taxonomy to categorize its diverse research and applications. This American Medical Extended Reality Association guideline, authored by the editorial board of the *Journal of Medical Extended Reality*, introduces a comprehensive taxonomy for MXR, developed through a multidisciplinary and international collaboration of experts. The guideline seeks to standardize terminology, categorize existing work, and provide a structured framework for future research and development in MXR. An international and multidisciplinary panel of experts was convened, selected based on publication track record, contributions to MXR, and other objective measures. Through an iterative process, the panel identified primary and secondary topics in MXR. These topics were refined over several rounds of review, leading to the final taxonomy. The taxonomy comprises 13 primary topics that jointly expand into 180 secondary topics, demonstrating the field's breadth and depth. At the core of the taxonomy are five overarching domains: (1) technological integration and innovation; (2) design, development, and deployment; (3) clinical and therapeutic applications; (4) education, training, and communication; and (5) ethical, regulatory, and socioeconomic considerations. The developed taxonomy offers a framework for categorizing the diverse research and applications within MXR. It may serve as a foundational tool for researchers, clinicians, funders, academic publishers, and regulators, facilitating clearer communication and categorization in this rapidly evolving field. As MXR continues to grow, this taxonomy will be instrumental in guiding its development and ensuring a cohesive understanding of its multifaceted nature.

## Introduction

Medical extended reality (MXR) represents a transformative intersection of health care and immersive technologies, encompassing virtual reality (VR), augmented reality (AR), mixed reality (MR), and other technologies that extend or enhance the medical experience.^1,2^ The roots of MXR trace back to the early experiments with VR in the 1980s and 1990s for psychological therapies and simulation training, among other uses.^3^

Over the years, as technology advanced, MXR applications broadened considerably, eventually gaining recognition from regulatory bodies such as the U.S. Food and Drug Administration (FDA).^4^ This acknowledgment by regulators not only marks an important milestone in the field of MXR but also paves the way for further innovation and integration into standard health care practices.

**FIG. 1.**
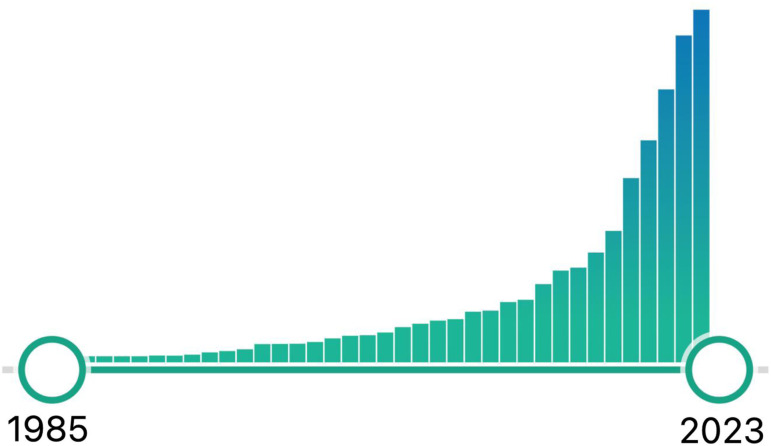
Annual mentions of the term “Virtual Reality” on PubMed: January 1985 through December 2023. There is an exponential rise in research, with >26,000 articles in the peer-reviewed literature as of the time of this article's publication.

The field of MXR has witnessed an exponential increase in scholarly interest, with tens of thousands of articles published, demonstrating year-over-year growth. Figure 1 illustrates the trajectory of peer-reviewed articles in PubMed that include the terms “virtual reality,” “augmented reality,” or “mixed reality”; it reveals an explosive increase in the MXR literature. As of this writing in January 2024, there have been >26,000 articles in academic science and medicine using these keywords.

The burgeoning body of MXR literature covers a vast array of topics, ranging from therapeutic domains (e.g., pain management,^5,6^ rehabilitation,^7,8^ and psychological interventions^9–11^) to procedural training,^12,13^ to medical education,^14^ to the ethics and acceptance of immersive systems, to the technological building blocks of MXR, such as combining artificial intelligence (AI) with spatial computing.^15,16^ This diversity reflects the versatility of MXR in addressing various health care needs, indicating its significant impact across multiple domains of medicine as well as the complexity of its interdisciplinary development and deployment.

Despite MXR's maturity beyond a nascent field, with significant advances in MXR research methodology,^17–21^ there remains a notable absence of a centralized taxonomy to categorize the diverse array of research and application areas within this domain. A well-structured taxonomy is important for facilitating clear communication and classification among different MXR participants, including researchers, grant funders, regulatory bodies, industry, and practitioners, among others. It serves as a foundational tool for organizing the field, advancing relevant theory, guiding future research directions, and fostering a cohesive understanding of MXR's multifaceted nature.

In response to this gap, the American Medical Extended Reality Association (AMXRA), in partnership with the editorial board of the *Journal of Medical Extended Reality*, developed a novel taxonomy to help define the field of MXR. Developed by experts in the field, this taxonomy reflects a comprehensive and multidisciplinary perspective that seeks to capture the current breadth and depth of MXR. It aims to standardize terminology, categorize existing work, and provide a framework for future research and development within this dynamic and rapidly evolving field.

## Methods

In developing the taxonomy for MXR, our approach was to be both multidisciplinary and international, engaging a diverse group of experts to contribute their insights and expertise. The selection of these members was based on several criteria, reflecting their impact and contributions to the field. This included:

- Publication track record: Preference was given to individuals with a substantial number of publications in MXR-related areas.
- Contributions to MXR: We sought experts who had made significant practical or theoretical contributions to the field.
- International perspectives: Recognizing the global scope of MXR, experts from different geographical regions were included to ensure a wide array of cultural and regional insights.
- Multidisciplinary perspectives: Given the interdisciplinary nature of MXR, individuals from diverse fields including psychology, communication, medicine, surgery, pediatrics, computer science, education, AI, design, architecture, engineering, biomedical imaging, simulation, education, and digital health were included.
- Other objective measures: Additional criteria such as leadership roles in professional societies, awards, and participation in prominent MXR projects were also considered.

The full list of participating members includes the 16 primary authors of this article together with the 13 individuals listed in the acknowledgment section who provided support in reviewing the taxonomy. The assembled team embarked on a structured, iterative process to develop the taxonomy. Initially, each member was asked to identify core topics within MXR from their unique perspective. This step generated a starting list of primary topics for subsequent review.

Next, a second round of review was conducted where the entire group was invited to evaluate the compiled list. This phase involved adding new topics that were missed in the initial round, consolidating topics that appeared overly similar, and separating those that were too heterogeneous. In addition, the primary author posted the draft taxonomy online using X (formerly Twitter) to solicit additional feedback from the general community. This iterative process sought to ensure a thorough and inclusive approach to capturing the breadth of MXR in medical science and practice.

In the third and final round, the focus shifted to fine tuning. The full group was given the opportunity to review and refine the topic names and ensure accurate categorization of the secondary topics. This process led to the development of the taxonomy presented in this article.

The resulting taxonomy is not meant to be static. MXR, like any dynamic field of science and technology, continues to evolve. Therefore, the taxonomy developed here should be viewed as a current snapshot, with the understanding that it will require updates and revisions as the field progresses and new insights emerge.

## Results

Table 1 presents the developed MXR taxonomy. This table delineates the 13 primary MXR topics identified by the panel, each further divided into a total of 180 granular secondary topics. These topics seek to encompass the current scope of MXR, demonstrating its extensive range and diverse applications. The Supplementary Appendix SA1 provides information on the round-by-round edits to the taxonomy. In the following sections, we provide a narrative description of each primary MXR topic.

**Table 1.** Taxonomy of Primary and Secondary Topics Encompassing the Field of Medical Extended Reality

1. XR hardware and sensors • Head-mounted displays • Tracking and positional systems • Haptics in XR • Input devices for XR • Sensory integration in XR • Biometric sensors in XR • Eye tracking and gaze interaction • Spatial computing platforms • Wireless communications in XR • Power and energy efficiency • Wearable XR technology • Medical and health care sensors in XR • Advanced display technologies • Sensor calibration and accuracy • Future trends and innovations in XR hardware and sensors • Other XR hardware and sensor 2. XR in medical imaging • Medical data visualization • XR for medical data overlay • Multimodal medical image fusion in XR • Holographic display of medical images • Holography for interactive medical data exploration • XR-enhanced navigation during interventional procedures • Other XR application in medical imaging 3. AI in MXR • AI agents • Automated reasoning • Machine learning • Natural language processing • Robotics • Computer vision • Reinforcement learning • Other AI in MXR 4. MXR design, development, and clinical deployment • Human-centered design in MXR • User interface/user experience • Clinician workflow • Human–data interactions • Decision support systems • Stakeholder adoption • Interoperability • Data management • Clinical informatics • Operational deployment • Other MXR design, development, or deployment 5. XR neuropsychological and biological mechanisms of action • Biofeedback in XR • Biophilia/nature environments • Distraction/spotlight of attention • Meditation effects of XR • Effects multisensory input in XR (e.g., spatial audio, olfaction, haptic/tactile) • Immunological effects of XR • Neurobiological effects of XR • Placebo/sham effects • Presence/immersion • Physiological effects of XR • Proteus effect • Alterations of time perception • Virtual embodiment • Other mechanism of action 6. Clinical applications of XR in neurology, psychology, or neurosensory modulation • XR for pain management • XR for neurodevelopmental disorders • XR for neurocognitive disorders • XR for neurorehabilitation • XR for movement disorders • XR for functional neurological disorders • XR in ophthalmology and optometry • XR for schizophrenia spectrum and other psychotic disorders • XR for bipolar and related disorders • XR for depressive disorders • XR for anxiety disorders • XR for obsessive-compulsive and related disorders • XR for trauma- and stressor-related disorders • XR for dissociative disorders • XR for somatic symptom and related disorders • XR for feeding and eating disorders • XR for sleep–wake disorders • XR for sexual dysfunctions • XR for gender dysphoria • XR for disruptive, impulse-control, and conduct disorders • XR for substance-related and addictive disorders • XR for personality disorders • XR for social isolation or loneliness • Other neuropsychological application 7. Clinical applications of XR in nonsurgical specialties (e.g., medicine, pediatrics, dentistry, nursing) • XR for cancer care and infusion • XR for cardiovascular disorders • XR in physical medicine and rehabilitation • XR in sports medicine • XR for digestive disorders • XR for hematological disorders • XR for infectious diseases • XR for endocrine disorders • XR for rheumatological disorders • XR for pulmonary disorders • XR for dermatological disorders • XR in emergency medicine • XR in intensive or critical care • XR in palliative care • XR in allergy and immunology • XR in medical genetics • XR in pathology • XR in pediatrics • XR in geriatrics • XR in nursing • XR in dentistry • XR in military and space medicine • Other nonsurgical applications of XR 8. Clinical applications of XR in perioperative and surgical specialties • XR in anesthesiology • XR in cardiothoracic surgery • XR in gastrointestinal surgery • XR in ophthalmic surgery • XR in plastic and reconstructive surgery • XR in hand surgery • XR in maxillofacial surgery • XR in surgical oncology • XR in surgical critical care • XR in transplant surgery • XR in trauma surgery • XR in general surgery • XR in neurosurgery • XR in orthopedic surgery • XR in urological surgery • XR in vascular surgery • XR in otolaryngology • XR for perioperative management • XR in obstetrics and gynecology • XR in podiatry • Other surgical application of XR 9. Therapeutic applications of XR in allied health services • XR in speech and language pathology • XR in occupational therapy • XR in midwifery • XR in hospice care • XR in audiological assessments and interventions • XR in dietary and nutritional counseling • XR in respiratory therapy sessions • XR tools for visual and orthoptic assessments • XR in chiropractic adjustments and patient education • XR for athletic and sports injury rehabilitation • XR in dental hygiene training and patient education • XR in genetic counseling sessions • XR in community health outreach and education • XR in prehospital simulations and emergency response training • XR for prosthetic and orthotic design and training • Patient-centered virtual environments in allied health • Other XR applications in allied health services 10. XR for patient education and communication • Telemedicine and remote XR consultations • Visualizing treatment options • Enhancing patient engagement • Cultural sensitivity and XR • XR as a support tool for caregivers • Feedback and patient input in XR • Patient-centered design thinking • Other XR application for patient education 11. XR for medical or surgical training and education • Virtual surgical or procedural simulators • Procedural training in AR • Anatomic exploration and education in XR • XR simulation for diagnostics • Patient interaction simulation • Team-based training in XR • Patient safety simulations • Surgical planning and rehearsal • Remote training and telemedicine • Assessment and competency evaluation • Long-term skill retention • Other XR application for medical education or training 12. Ethics, safety, privacy, and adverse effects of MXR • Cybersickness/simulator sickness • Visual adverse effects • Neurological adverse effects • Musculoskeletal and postural adverse effects • Skin irritation/dermatological adverse effects • Psychological adverse effects • Injury risk • Negative impacts on children/adolescents • XR overuse or addiction • Social isolation • Ethical considerations • Data privacy and security • Certification of clinician expertise in using MXR • Other safety and adverse effects of MXR 13. Socioeconomic and regulatory aspects of MXR • Regulatory oversight of MXR • Research methodology in MXR • Health equity in MXR • Policy and payment of MXR • Health economics of MXR • Legal considerations of MXR • Other socioeconomic or regulatory

AI, artificial intelligence; AR, augmented reality; MXR, medical extended reality.

### Hardware and sensors

This section focuses on the technological aspects of XR, including head-mounted displays, tracking systems, haptics, input devices, and sensory integration. It encompasses advancements in biometric sensors, spatial computing, wireless communications, and wearable technologies essential for XR's functionality, fidelity, and data inputs, among other related topics.

### Medical imaging

Focusing on XR's role in medical imaging, this area covers medical data visualization, image fusion, holographic imaging, and interactive data exploration. It also includes XR-enhanced navigation for interventional procedures, highlighting XR's potential to transform traditional imaging methods.

### Artificial intelligence

Here, the focus is on integrating AI with MXR to enhance application capability in areas such as medical decision making and treatment tailoring. Topics include AI agents, AI medical simulations, machine learning, natural language processing, and robotics within the context of MXR technologies, underscoring the synergistic potential of AI and XR in health care.

### Design, development, and clinical deployment

This topic delves into building blocks of MXR applications and the design and development aspects of MXR, including user interface, clinician workflow, decision support systems, and stakeholder adoption. It also covers interoperability, clinical informatics, data management, and intervention science topics that underlie operational deployment, essential for the practical application of MXR.

### Neuropsychological and biological mechanisms of action

This topic focuses on how MXR technologies influence psychological and biological processes and encompasses their underlying mechanisms of action. It covers subtopics such as biofeedback in XR, the psychological influence of biophilic nature environments, modifying the spotlight of attention, sensory and psychological effects of multisensory input in XR (e.g., spatial audio, olfaction, and haptic/tactile), and the immunological and neurobiological effects of XR use. It also delves into psychological topics such as presence and immersion, time modification in XR, the Proteus effect, virtual embodiment, and how to address these features in research design.

### Clinical applications in neurology, psychology, psychiatry, or neurosensory modulation

This area focuses on using, evaluating, and understanding XR for treating neurological disorders, psychological conditions, and supporting neurosensory modulation to help manage acute and chronic pain. It includes applications in managing neurodevelopmental, neurocognitive, and movement disorders, neurorehabilitation, and various mental health conditions, offering alternative approaches to traditional therapeutic methods.

### Clinical applications in nonsurgical specialties

This section encompasses topics related to XR's role in nonsurgical specialties, including medicine, pediatrics, dentistry, and nursing. It covers a wide range of applications including oncology, hematology, cardiology, gastroenterology, endocrinology, and infectious diseases, among others. It also includes applications in occupational medicine, sports medicine, geriatrics, military medicine, and space medicine, demonstrating XR's versatility in different medical contexts across the lifespan and in varied treatment settings.

Pediatric-specific topics include developmental considerations for MXR, pediatric training and education, pediatric-friendly interface and design, and ethical considerations and child protection, among others. In aging populations, additional topics include MXR for neurocognitive decline, addressing social isolation, and end-of-life care.

### Clinical applications in perioperative and surgical specialties

Here, the focus is on XR applications across various perioperative and surgical specialties, including anesthesiology, cardiothoracic, gastrointestinal, urological, ophthalmic, obstetrics, plastic surgery, and podiatric, among others. It explores how XR can enhance perioperative management, surgical planning, training, and execution, as well as issues around use of XR in the surgical environment.

### Therapeutic applications in allied health services

This topic explores XR applications in allied health services such as speech and language pathology, occupational therapy, midwifery, nutrition, hospice care, out-of-hospital medicine (e.g., paramedical services), and more. It covers how XR can be used in patient education, physical rehabilitation, and various other therapeutic contexts, highlighting its role in supporting and enhancing the effectiveness of allied health services.

### Patient education and communication

This area explores XR's applications in patient education and communication, including telemedicine in XR, treatment visualization and tailoring, patient engagement, immersive health educational tools, XR tools for patient and consumer self-care, and support for caregivers. It highlights how XR can bridge communication gaps and enhance patient-centered care in a culturally sensitive and effective manner.

### Health care professional training and education

This section examines XR's impact on health care professional education and training, including virtual simulators, procedural training, anatomical exploration, remote training in XR, and patient interaction and safety simulations. It emphasizes XR's role in enhancing educational methodologies and training effectiveness in health care settings.

### Harm reduction

This domain encompasses a comprehensive approach to minimizing the risks and enhancing the safe use of XR technologies. Harm reduction within MXR is multifaceted, addressing a spectrum of concerns from user safety to ethical practices. It includes managing direct hazards such as cybersickness, visual and neurological adverse effects, musculoskeletal issues, and injury risk. The domain also considers psychological and social well-being, focusing on the potential for mental health impacts, as well as the risks of overuse and dependency on XR technologies.

Ethical considerations are pivotal, including equitable access, safeguarding data privacy, maintaining security, and ensuring the authenticity of personal identity within XR environments. The commitment to harm reduction is particularly critical for protecting vulnerable and marginalized populations, ensuring that the advancement of MXR technologies does not exacerbate disparities but instead contributes to a more inclusive and safe health care ecosystem.

### Socioeconomic and regulatory aspects

Focusing on the broader context of MXR, this topic explores regulatory oversight, health equity, policy, health economics, and legal considerations. It underscores the importance of creating a conducive social, economic, and regulatory environment for MXR's growth, access and protection for patients and communities, and integration into health care systems.

**FIG. 2.**
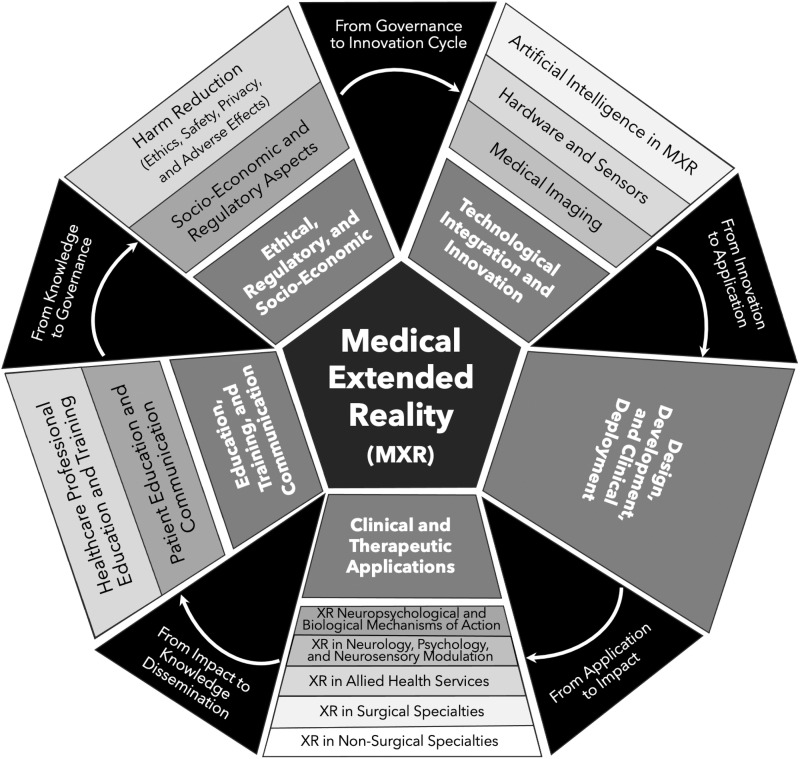
Visual depiction of the MXR taxonomy. At its core, MXR encompasses five broad domains. These core domains expand into 13 primary topics, which then explode into the 180 secondary topics presented in Table 1. See text for more information about the emergent narrative depicted by the transition phrases between domains. MXR, medical extended reality.

Figure 2 offers a visual representation of the MXR taxonomy, illustrating how the field's various components interconnect. At the core are five overarching domains, which serve as the foundational and linking themes for MXR. These include (1) technological integration and innovation; (2) design, development, and deployment; (3) clinical and therapeutic applications; (4) education, training, and communication; and (5) ethical, regulatory, and socioeconomic considerations.

From these central themes, the diagram branches out to the 13 primary topics identified in the taxonomy, each depicted in the outer rings. This layout provides a hierarchical view of MXR, from broad thematic areas to specific research and application topics, encapsulating the full scope and structure of the field as it currently stands.

Beyond the individual domains, primary topics, and secondary topics, the figure indicates a cohesive narrative that captures the lifecycle and governance of this field. This relationship is visually articulated in Figure 2, which outlines an emergent narrative connecting the five core domains.

This narrative is inherently cyclical and interwoven but can begin with the “Technological and Innovation” domain, which is the ever-evolving bedrock of MXR. This domain is dedicated to the technological advancements and innovative tools that propel MXR forward and enable its therapeutic and educational potential. It encapsulates cutting-edge research and development pushing the boundaries possibility in MXR and its integration with other health care technologies.

Progressing from this foundational domain, we move into “Design, Development, and Deployment.” This transition, encapsulated by the phrase “From Innovation to Application,” represents the crucial phase where technological breakthroughs are aligned with health care needs and end-user perspectives, and harnessed to create practical tools for health care. It is here that the conceptual becomes tangible, leading to MXR solutions that can be deployed in clinical settings.

The narrative then unfolds into “Clinical and Therapeutic Applications,” where the tangible applications of MXR come to life. Under the transition labeled “Application to Impact,” we see the direct influence of MXR in health care delivery and engagement in evaluation and optimization of MXR applications. Here, design and development efforts manifest in the form of therapeutic interventions and clinical applications.

Next, we consider the domain of “Education, Training, and Communication,” linked by the notion of “Impact to Knowledge Dissemination.” The experiences and outcomes derived from clinical applications inform educational content and strategies, enhancing the training of health care professionals. Furthermore, this domain emphasizes the significance of leveraging MXR developments and impact for patient communication and education, ensuring that the benefits of MXR technologies are fully realized in their capability for effective knowledge translation and conveyance.

Finally, we arrive at the “Ethical, Regulatory, and Socio-Economic” domain, bridged by the phrase “Knowledge to Governance.” This segment of the narrative focuses on how the insights gained through education and practice inform the ethical frameworks and regulatory guidelines that govern the use of MXR. Moreover, it touches upon the interface between socioeconomic systems and MXR deployment, considering the broader impact on health care systems and society.

This narrative structure not only highlights the individual importance of each domain but also the essential connections between them. The figure serves as a visual representation of this narrative, illustrating the flow from one domain to the next, reflecting a synergistic ecosystem within MXR. Of note, research and systematic evaluation are integral components at every stage of this narrative, permeating each domain, primary topic, and secondary topic. They act as the driving force behind the continual advancement of MXR, propelling the field's growth and innovation.

## Discussion

The development of a taxonomy for MXR is an important step in supporting the field and its diverse stakeholders. By establishing a structured classification of topics and concepts, this AMXRA guideline provides a framework for the categorization and understanding of MXR research and applications. With this study, we have formulated an initial taxonomy with 5 core domains, encompassing 13 primary topics and a comprehensive set of 180 secondary topics. This taxonomy offers a tool for researchers, practitioners, funders, industry, and other groups to systematically classify and navigate the complexities of the MXR field.

MXR is an inherently dynamic and evolving field, and as such, no taxonomy can claim to be definitive or exhaustive. Our approach involved input from experts with extensive knowledge of the literature to identify the most relevant primary and secondary topics. However, there may be emerging or unrepresented topics that are not included in the current schema. We view this taxonomy as a “living document,” open to modifications and expansions in response to new developments and insights in the field. This adaptability ensures that the taxonomy remains relevant and reflective of the ongoing advancements in MXR.

There are many potential applications for this taxonomy. For example, it will be employed to classify research submitted to the *Journal of Medical Extended Reality*, illustrating one of its practical uses. The naming system may also be useful to other journals accepting MXR research. Beyond academic publishing, it can serve as a guide for regulators to categorize MXR applications, assist research funders in identifying pertinent areas of focus, and provide a common language for the broader MXR community.

MXR, as a distinct and burgeoning field, is set to expand further with technological advancements. This taxonomy, therefore, not only defines the current landscape of MXR but is also designed to evolve and grow alongside the field itself, underlining its enduring relevance and utility in shaping the future of health care technology.

## Supplementary Material

Supplementary Appendix SA1

## Abbreviations Used

AI: artificial intelligence
AMXRA: American Medical Extended Reality Association
MXR: medical extended reality
