# Supplementary material for: What Is Medical Extended Reality? A Taxonomy Defining the Current Breadth and Depth of an Evolving Field
Source: J Med Ext Real. 2024 Jan 25;1(1):4–12. doi: 10.1089/jmxr.2023.0012 (PMC10945763; doi:10.1089/jmxr.2023.0012)
Supplement: Supplemental data [file Suppl_AppendixSA1.docx]

**Supplementary Appendix**

The AMXRA panel developed the MXR taxonomy over the course of three rounds of development and review. In this supplementary appendix, additional details are provided about the results of each round, culminating in the final taxonomy presented in the accompanying guideline document.

**Round 1**

In round 1, initial topics were collected from members of the panel. The following list was compiled, intended to provide an initial view of the breadth and depth of MXR, here presented in alphabetical order.

| - Advanced Visualization Systems |
| --- |
| - Artificial Intelligence in MXR |
| - Biofeedback |
| - Ethics |
| - Hardware and Sensors |
| - Health Economics of MXR |
| - Informatics |
| - Mental Health Disorders |
| - MXR Regulation and Policy |
| - Pain Management |
| - Physical Therapy & Rehab |
| - Psychology & Psychometrics |
| - Virtual Embodiment |
| - XR for Cancer Care and Infusion |
| - XR for Cardiovascular Disorders |
| - XR for Dermatological Disorders |
| - XR for Digestive Disorders |
| - XR for Infectious Diseases |
| - XR for Medical Interview Training |
| - XR for Musculoskeletal Disorders |
| - XR for Neurological Disorders |
| - XR for Obstetrics & Gynecology |
| - XR for Palliative Care |
| - XR for Pediatrics |
| - XR for Pulmonary Disorders |
| - XR for Surgical or Procedural Training |
| - XR in Emergency Medicine |
| - XR in Intensive Care |
| - XR in Pharmacology |

**Round 2**

In round 2, the list of initial topics was expanded and organized into 9 primary topics and 109 secondary topics based upon input from 29 participating experts.

**1.**    **MXR Neuropsychological Mechanisms of Action (MOA)**

•         Biophilia / nature environments

•         Distraction / spotlight of attention

•         Time acceleration

•         Virtual embodiment

•         Proteus effect

•         Biofeedback

•         Presence

•         Placebo / sham effects

•         Spatial audio in XR environments

•         Other MOA

**2.**    **Therapeutic Applications of XR in Psychology, Neurology, Rehabilitation, or Pain Management**

- XR for acute pain
- XR for chronic pain
- XR for neurodevelopmental disorders
- XR for neurocognitive disorders
- XR for neurorehabilitation
- XR for musculoskeletal rehabilitation
- XR for movement disorders
- XR for schizophrenia spectrum and other psychotic disorders
- XR for bipolar and related disorders
- XR for depressive disorders
- XR for anxiety disorders
- XR for obsessive-compulsive and related disorders
- XR for trauma- and stressor-related disorders
- XR for dissociative disorders
- XR for somatic symptom and related disorders
- XR for feeding and eating disorders
- XR for sleep-wake disorders
- XR for sexual dysfunctions
- XR for gender dysphoria
- XR for disruptive, impulse-control, and conduct disorders
- XR for substance-related and addictive disorders
- XR for personality disorders
- Other psychological application

**3.**    **Therapeutic Applications of XR in Medicine, Pediatrics, Obstetrics/Gynecology, or Surgery**

- XR for digestive disorders
- XR for infectious diseases
- XR for cardiovascular disorders
- XR for cancer care and infusion
- XR for rheumatological or musculoskeletal disorders
- XR for pulmonary disorders
- XR in emergency medicine
- XR in intensive or critical care
- XR for perioperative management
- XR in palliative care
- XR in dermatological disorders
- XR in pediatrics
- XR in obstetrics
- XR in gynecology
- Other medical or surgical application

**4.**    **XR in Medical Imaging**

- Medical data visualization
- XR for medical data overlay
- Multimodal medical image fusion in XR
- Holographic display of medical images
- Holography for interactive medical data exploration
- XR-enhanced navigation during interventional procedures
- Other XR application in medical imaging

**5.**    **XR for Medical or Surgical Training and Education**

- Virtual surgical or procedural simulators
- Procedural training in AR
- Anatomic exploration and education in XR
- XR simulation for diagnostics
- Patient interaction simulation
- Team-based training in XR
- Patient safety simulations
- Surgical planning and rehearsal
- Remote training and telemedicine
- Assessment and competency evaluation
- Long-term skill retention
- Other XR application for medical education or training

**6.**    **XR for Patient Education and Communication**

- Telemedicine and remote XR consultations
- Visualizing treatment options
- Enhancing patient engagement
- Cultural sensitivity and XR
- XR as a support tool for caregivers
- Feedback and patient input in XR
- Patient-centered design thinking
- Other XR application for patient education

**7.**    **Artificial Intelligence, Informatics, Security, and Data Processing in MXR**

- AI agents
- Automated reasoning
- Machine learning
- Natural language processing
- Robotics
- Decision support systems
- Big data analytics
- Data privacy and security
- Human-data interactions
- Task analysis
- Interoperability
- Other AI, informatics, or data processing

**8.**       **XR Hardware and Sensors**

- Head-mounted displays
- Tracking and positional systems
- Input devices for XR
- Sensory integration in XR
- Biometric sensors in XR
- Eye tracking and gaze interaction
- Spatial computing platforms
- Wireless communications in XR
- Power and energy efficiency
- Wearable XR technology
- Medical and healthcare sensors in XR
- Advanced display technologies
- Sensor calibration and accuracy
- Future trends and innovations in XR hardware and sensors
- Other XR hardware and sensor

**9.**    **Socio-Economic, Ethical, or Regulatory Aspects of MXR**

•         Regulatory oversight of MXR

•         Health equity in MXR

•         Policy and payment of MXR

•         Health economics of MXR

•         Legal considerations of MXR

•         Ethical considerations of MXR

•         Other socio-economic, ethical, or regulatory

**Round 3**

In round 3, the updated list of topics was refined, yielding the final list of 13 primary topics and 180 secondary topics presented in the paper. There were 22 experts who provided input during this final round of review.
